# Clinical research in Brazil

**DOI:** 10.1590/S1679-45082013000100001

**Published:** 2013

**Affiliations:** 1 Hospital Israelita Albert Einstein, São Paulo, SP, Brazil

Clinical research is extremely important to advance medical care. It is impossible to predict how a drug will perform in humans given their complexities, based only on *in vitro* and animal studies. On the other hand protection of research subjects is not only a scientific question but one of human rights, vis-a-vis the recent past when abuses were committed even in the so-called civilized societies.

The quagmire between human research subjects and the performance of clinical trials is yet to be fully resolved and the best science can offer are guidelines and safety of a review made by ethics committees comprising not only peers, but also lawyers, representatives of patient groups and consumer advocates.

Unfortunately, the Brazilian authorities are positioning the country in such a way that soon clinical research will not be any longer possible here. The inadequacies of the centralized oversight of clinical research, notably those with international participation, present an important constraint in carrying out clinical studies in the country and leaves Brazil off the loop in the medical science community. The net result is Brazilian patients are excluded from the POSSIBILITY of a live-saving or life-altering drug until it is approved in other markets, sometimes many years after the fact. Also, Brazilian healthcare providers have no time to gain experience about the usage of the new drugs, and about some relevant issues, such as how to deal with the inevitable side effects. It gets worse, since you cannot have access to these drugs during the research phase. The peculiarities of the drug behavior in the Brazilian population are collected not under a controlled scientific environment but in the chaotic day-to-day life of physicians and healthcare organizations that are primary providers and not scientists. The result is that data are not acquired properly and timely as it would if research protocols were conducted.

Add to all of that, the regulatory nightmare of Brazilian bureaucracy, with multiple agencies involved in the definition of what may be brought to the country. Recently, in the tragedy in Santa Maria, medications had to be delivered from the United States to treat smoke-poisoned patients because the drug is not yet “allowed” in Brazil.

When compared to basic research, clinical studies are more complex and more expensive. Small, local and regional studies, including complex clinical trials as well as epidemiologic studies, have a limited impact on clinical practice, demanding national or global networks. Hence, logistics, mainly for clinical trials, requires a complex structure. Global recruitment, well-organized clinical research units with full acknowledgement of good clinical practices, long-term monitoring and the need to provide “beneficial” experimental drugs after the trial, increase the costs so much that these clinical trials involving drug treatment and new interventions are today almost exclusively conducted by large pharmaceutical companies. As a result, research units dedicated to conduct global studies are having problems with budgets, mainly in developing countries.

And problems continue after publication of good results. The threats of manipulated data studies, mainly those (but not only) conducted by big pharmaceutical companies, are emerging. Comply and control mechanisms are lacking.

Another difficulty in the mastering role of pharmaceutical companies in clinical research is the fact that new compounds not always meet the needs of patients and clinicians. Let's take the example of antibiotics: no new drugs are expected in the short term for Gram-negative bacteria (a group of bacteria with species resistant to virtually all antibiotics); there are no such medications under development since the industries considered them not profitable enough. Infectious disease specialists are used to advocate restrictions for wide antibiotic use to preserve their efficacy. The use restrictions denotes decrease of rentability in order to develop these drugs.

The pharmaceutical industries, however, play an important role in discovering and developing new drugs. Recent therapeutic approaches, despite some unexpected findings after clinical studies with a large number of patients treated, have changed clinical practice and saved a lot of lives. New anticoagulants, antibacterial, antifungal, anti-inflammatory and oncological drugs have made clinical practice easier and increased patient safety. Working together with large pharmaceutical companies, rather than decrease their role in researchers, should be the best strategy to have new good clinical trials. Lessening bureaucratic barriers and costs and elaborating policies for compliance with control practices, mainly regarding data analysis, collection and manuscript elaboration are key for a symbiotic relationship.

Epidemiologic and translational studies are also relevant. They are less expensive and private and public funding should be encouraged, understanding that they are the predecessors of large clinical trials.

Clinical studies are of direct benefit for patients. In some areas (oncology, inflammatory disease) patients participate of clinical trials of new drugs because no other option is available for their diseases. We have so many other problems that the “details” of clinical research are much overshadowed and average citizens cannot perceive the damage caused to them by those who should work to their favor. It is therefore the obligation of all professionals in the area, physicians, pharmacists, nurses, biologists – to name a few, to organize and propose a rational approach that will not only quench the regulatory thirst of the government but will also serve patients.

